# A biophysical perspective on receptor-mediated virus entry with a focus on HIV

**DOI:** 10.1016/j.bbamem.2019.183158

**Published:** 2020-06-01

**Authors:** Isabel Llorente García, Mark Marsh

**Affiliations:** a Dept. of Physics and Astronomy, University College London, London, UK; b Medical Research Council Laboratory for Molecular Cell Biology, University College London, London, UK

**Keywords:** Virus entry, HIV, Biophysics, Virus-receptor interactions, Membrane receptor properties

## Abstract

As part of their entry and infection strategy, viruses interact with specific receptor molecules expressed on the surface of target cells. The efficiency and kinetics of the virus-receptor interactions required for a virus to productively infect a cell is determined by the biophysical properties of the receptors, which are in turn influenced by the receptors' plasma membrane (PM) environments. Currently, little is known about the biophysical properties of these receptor molecules or their engagement during virus binding and entry. Here we review virus-receptor interactions focusing on the human immunodeficiency virus type 1 (HIV), the etiological agent of acquired immunodeficiency syndrome (AIDS), as a model system. HIV is one of the best characterised enveloped viruses, with the identity, roles and structure of the key molecules required for infection well established. We review current knowledge of receptor-mediated HIV entry, addressing the properties of the HIV cell-surface receptors, the techniques used to measure these properties, and the macromolecular interactions and events required for virus entry. We discuss some of the key biophysical principles underlying receptor-mediated virus entry and attempt to interpret the available data in the context of biophysical mechanisms. We also highlight crucial outstanding questions and consider how new tools might be applied to advance understanding of the biophysical properties of viral receptors and the dynamic events leading to virus entry.

## Highlights

• Our lack of detailed understanding of the biophysical properties of HIV receptors
• The contradictory nature of the published data for CD4 and CCR5 (e.g. diffusion)
• A set of clear conditions that is needed to compare experiments
• Preferred techniques and crucial outstanding questions

## Graphical abstract

**Figure:**
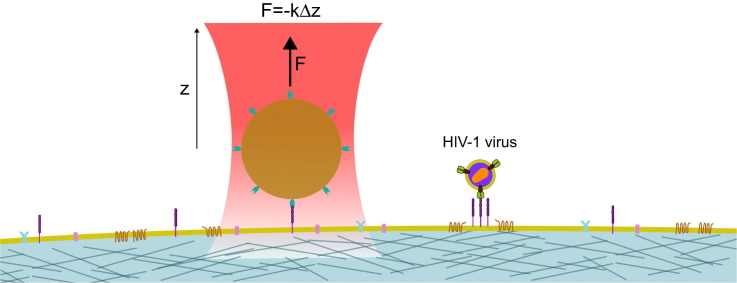

## 1 Introduction

### 1.1 Background

**Fig. 1:**
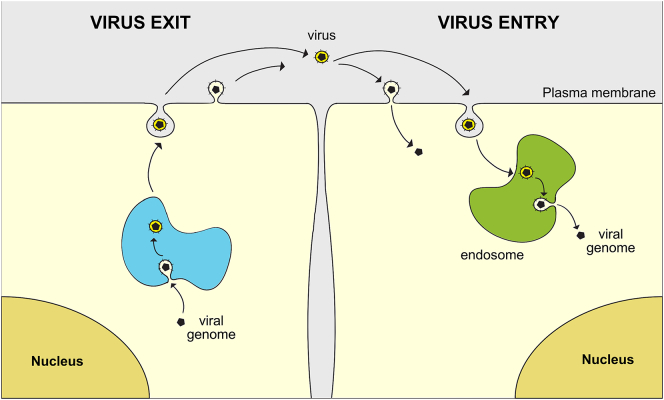
Enveloped virus life cycle. Left: Viral nucleic acid and proteins produced in an infected cell are assembled into membrane-containing infectious particles that are released from the cell. For some viruses, e.g. Flaviviruses (Zika, Dengue), assembly occurs on intracellular membranes. For many others, including HIV, assembly occurs at the plasma membrane. Right: Enveloped virus penetrate new host cells by membrane fusion, either directly at the plasma membrane or following endocytic uptake into endosomes. Both processes lead to the release of nucleic acid-containing viral cores into the cytoplasm, events that subsequently lead to uncoating, cellular infection and viral replication though mechanisms that vary for different viruses.

Viruses can be classified as either enveloped or non-enveloped. For enveloped viruses, such as HIV, the genetic material and core proteins are contained within a lipid membrane, the envelope, during the extracellular phase of a viral life cycle (Fig. 1). The envelope is derived from a host cell membrane, often the PM, during the assembly of new virions [1]. To enter new host cells, viruses must penetrate the barrier presented by the PM of the target cell. For enveloped viruses, this is achieved by membrane fusion, which can occur either at the cell surface or from within endocytic organelles following endocytosis of intact viruses (Fig. 1). Non-enveloped viruses, which lack membranes, use other penetration mechanisms that are not discussed here, though some aspects of receptor engagement are likely to be similar.

**Fig. 2:**
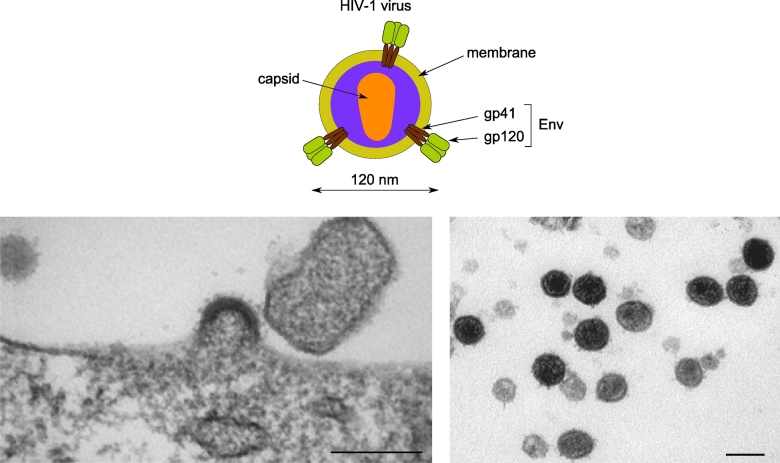
Top: Schematic of HIV structure showing the mature viral capsid, containing the viral RNA, and the viral membrane with embedded Env proteins. Bottom left: HIV particle assembly: Electron micrograph of an assembling HIV particle; the electron dense layer of p55Gag is clearly visible underlying the bilayer plasma membrane that contains multiple copies of the viral envelope glycoprotein, Scale bar: 200 nm. Bottom right: Electron micrograph of mature HIV particles, Scale bar: 150 nm. Images courtesy of Dr. Jemima Burden, MRC LMCB, UCL.

HIV is a lentivirus, a genus of viruses within the *retroviridae* family, that forms ~125 nm diameter spherical virions (Fig. 2) [1,2]. The viral membrane comprises a lipid bilayer and the essential virally-encoded envelope glycoprotein (Env). Env is the viral protein that engages cell surface receptors and mediates membrane fusion [3,4]. Each Env molecule is formed from three gp160 precursor transmembrane proteins that assemble into a trimer following synthesis on the rough endoplasmic reticulum (rER) of infected cells. Following initial folding and N-linked glycosylation, these trimers are transported, via the Golgi apparatus, to the PM. *En route*, each gp160 monomer is proteolytically cleaved to generate two non-covalently linked glycoproteins: 1) the extracellular gp120 [aka surface unit (SU)] and 2) the transmembrane gp41 [aka transmembrane (TM)] (Fig. 2). The external domain of each Env trimer appears as a spike that protrudes ~10 nm from the virion surface; each spike is 10–15 nm wide at its head (Fig. 2; [[5], [6], [7], [8], [9], [10], [11], [12], [13]]). Most enveloped viruses (e.g. influenza virus) contain many copies of their respective envelope glycoprotein(s). HIV is unusual in that it contains relatively few Env trimers (~10 per particle [7,14,15]) leaving space in the viral envelope for cellular membrane proteins which, though not essential for viral entry/replication, may aid viral binding to target cells and/or the kinetics of entry [16,17].

Regardless of their structure, all viruses must engage with specific receptors during entry into cells, and frequently it is the use of these receptors that determines tropism, i.e. the susceptibility of particular hosts and cell types to infection by a specific virus. Receptors are generally nanometre-sized protein molecules, or protein complexes, embedded in a cell's PM. Typically, they consist of an extracellular domain, which is accessible on the surface of intact cells, one or more transmembrane domains that span the lipid bilayer, and an intracellular domain(s) that project into the cytoplasm of the cell. In some cases protein receptors may be linked to the membrane by a lipid moiety [18,19], or receptors may be lipid molecules or sugar moieties attached to membrane glycoproteins or glycolipids. Cell-surface receptors normally have essential cellular functions that can include roles in nutrition, cell migration, immune recognition, adhesion and growth control. These functions frequently involve communication between the extracellular environment and a cell's interior. Thus, intracellular signalling can be triggered when ligands bind to the extracellular domain(s) of their cognate receptor initiating changes in the receptor's cytoplasmic domain(s). Changes in the cytoplasmic domain(s) may in turn affect the extracellular binding of ligands. For viruses, exploiting this signalling potential may be important for entry and subsequent replication [17,20,21].

Whether transmembrane domain-containing, or lipid-linked, virus receptor proteins undergo Brownian diffusion in the plane of the membrane. The physical interactions of receptor proteins with membrane lipids, and other membrane-associated proteins, influence these diffusion properties. However, we lack a clear understanding of how these interactions and properties, as well as variations in receptor density and membrane trafficking, affect the kinetics and affinity/avidity of virus-host cell engagement leading to productive infection.

### 1.2 HIV receptors

**Fig. 3:**
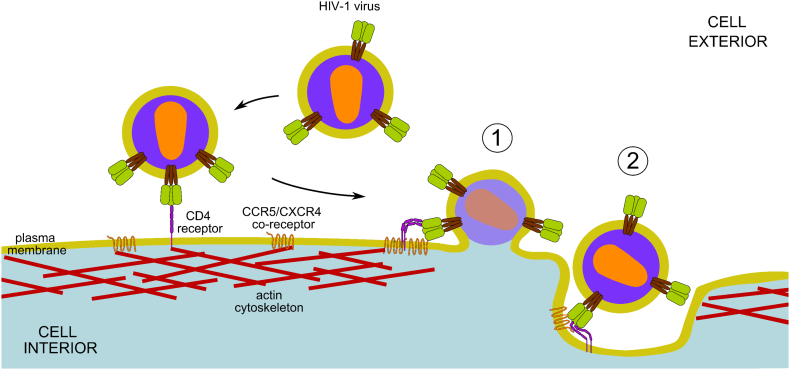
Schematic of HIV entry. HIV Env first interacts with cell surface CD4 molecules and subsequently with CCR5 and/or CXCR4 co-receptors, leading either to fusion at the plasma membrane (①) or endocytosis (②) followed by fusion from within endocytic vesicles.

The Cluster of Differentiation antigen 4 (CD4) and the co-receptors CC chemokine receptor 5 (CCR5) and/or CXC chemokine receptor 4 (CXCR4) have been identified as the principal receptors for HIV. CD4 is the primary receptor to which HIV Env binds. A co-receptor (CCR5/CXCR4) is subsequently engaged following the display of co-receptor binding sites. This later induces conformational changes in Env that lead to virus fusion and entry. Although virtually all HIV strains use CD4, the viruses that usually mediate primary infections use CCR5 as co-receptors and infect CCR5 expressing cells (so called R5 HIVs). By contrast, X4 HIV strains, which can be associated with late stage disease, use CXCR4 as co-receptors. Dual-tropic HIVs can infect CD4^+^ cells using either co-receptor. CD4, CCR5 and CXCR4 molecules are expressed on key cells of the immune system, i.e. CD4^+^ T lymphocytes and myeloid cells (dendritic cells and macrophages) - the primary targets for HIV infection and pathogenesis [4]. CD4 normally functions in T-cell activation events that contribute to adaptive immunity. In this context, CD4 has a signalling function, acting as a co-receptor, together with T-cell antigen receptors, for MHC class II molecules on antigen-presenting cells. CD4 is a type I integral membrane glycoprotein with four extracellular immunoglobulin (Ig)-like domains (D1-D4), each of which is about 3 nm in length (for comparison, the lipid bilayer is ~6 nm thick), a transmembrane domain and a short 40 amino acid cytoplasmic domain (Fig. 3) [22]. The outermost D1 Ig domain contains the binding site that engages HIV Env. By contrast, CCR5 and CXCR4 are members of the super-family of 7-transmembrane domain, G-protein-coupled receptors (GPCR) [[23], [24], [25]]. The chemokine receptor sub-group of GPCRs generally has a short extracellular amino-terminal domain, the obligatory 7 transmembrane domains linked by short peptide loops, and carboxy-terminal cytoplasmic domains that vary in length (Fig. 3). The amino-terminus and the second extracellular loop are implicated in HIV-gp120 binding. The overall dimensions of CCR5 monomers are ~ 5–10 nm [26]. Normally, these receptors function in chemotactic responses to small peptide ligands (chemokines) that regulate, for example, immune cell recruitment, vascularisation, axon outgrowth and perhaps memory [[27], [28], [29]].

During HIV entry, HIV Env gp120 engages CD4 molecules on the target cell [[30], [31], [32]] (Fig. 3). This interaction induces conformational changes in Env [[33], [34], [35]], or stabilises a conformational state [36,37], in which a co-receptor binding site is exposed enabling gp120 to bind CCR5 or CXCR4 [[38], [39], [40],^17^]. The engagement of gp120 with either co-receptor initiates further conformational changes in gp120 and gp41, as well as signalling functions, that drive fusion of the viral and cellular membranes [20,17,21]. A number of studies have indicated that HIV fusion occurs at the surface of target cells, and cell-surface fusion is usually considered the main route for HIV entry [41,42]. But fusion may also occur later, after bound virions have been internalised into endocytic vesicles [[43], [44], [45], [46], [47], [48], [49]]. The ability to exploit both pathways (Fig. 1) may enable HIV to infect cells in a range of different cellular contexts, receptor densities and binding conditions [42,44,50,51]. Regardless, membrane fusion is an essential feature of both mechanisms and leads to release of the viral capsid into the cytoplasm of the target cell, initiating viral replication and the pathogenic consequences of cellular infection. The typical time scale of the virus entry events from engagement to fusion is of the order of minutes. In vivo, HIV can exist as free virions and infection can occur when these virus particles engage new target cells. However, it is widely believed that direct cell-to-cell transmission also occurs, is 10–1000 fold more efficient than cell-free virus infection, and is likely to be of particular relevance to infection in cell-dense contexts such as lymphoid tissues [[52], [53], [54]]. Direct cell-to-cell transmission still requires the formation of virus particles, but these are retained within so-called virological synapses, regions of intimate contact between infected and uninfected cells [55,56]. The early infection events that are associated with person-person transmission typically occur in the mucosal barriers of the vagina or rectum, or in the mucosa of the penis prepuce (foreskin) or of the coronal sulcus and glans, and may involve cell-free virus interactions. By contrast, subsequent viral transmission occurs primarily in lymphoid tissues where direct cell-to-cell transfer may be more prevalent [57].

Other receptor molecules have been described, e.g. DC-SIGN, which mediates non-infectious uptake into dendritic cells prior to transfer of virus to T cells during trans-infection through virological synapses. Similarly, the glycolipid, galactocerebroside, has been demonstrated to bind Env [58]. Though these interactions may have some role in virus association with specific cells, CD4 and the co-receptor molecules CCR5 and CXCR4 remain the key receptors involved in viral fusion and cellular infection.

### 1.3 Plasma membrane organisation and influence of the cytoskeleton

The characteristics of all cell-surface receptors are strongly influenced by the properties of the membrane in which they are embedded (the same is true for Env in the virus membrane). In the aqueous environment in which cells exist, and at physiological temperatures (~37 °C), receptor molecules can diffuse in the plane of the membrane (inertia is negligible and viscous drag and random thermal Brownian fluctuations dominate). This diffusive mobility then determines the probability of individual receptor molecules undergoing homologous or heterologous interactions. However, receptor mobility is also influenced by other factors such as local lipid environments, receptor oligomerisation and receptor interactions with the cortical cytoskeleton, as detailed below. Here we limit our discussion to the PM. This key cellular membrane system is not homogeneous but is compartmentalised by dynamic interactions between its constituent lipids and proteins and by supporting structures such as the actin cortex. In addition to the vast number of different proteins, the PM contains numerous different lipid species many of which have some propensity to self-organise. Thus, local lipid domains with distinct compositions can determine local membrane fluidity. So-called ‘lipid raft’ nano-domains [59,60] [~2–20 nm in size, though sometimes larger [61]], enriched in cholesterol, saturated phospholipids and sphingolipids, exhibit increased lipid packing densities and reduced membrane fluidity. Lipid rafts can interact with subsets of transmembrane proteins or lipid-linked proteins and can thus affect the compartmentalisation, clustering, diffusive behaviour and trafficking of PM receptors [62]. By contrast, non-raft lipids, such as unsaturated phospholipids, form liquid disordered domains that exhibit increased fluidity and receptor mobility [61,63,64]. Receptor residency times in the PM, which are linked to internalisation into intracellular compartments and recycling to the PM, can also influence virus-receptor interactions, as does PM ruffling and protrusion (e.g. microvilli and blebs).

The cortical cytoskeleton, an actin-based structure that underlies and confers mechanical support to the PM, is a dense meshwork of proteins, up to 100 nm thick, comprised mainly of actin filaments (aka filamentous actin (F-actin): ~6 nm diameter and up to several μm long), myosin molecular motors, actin-binding proteins and other linker proteins [65] that connect the actin cortex to the PM to regulate its functionality. The cortex is highly dynamic: it can locally disassemble and reassemble, change shape, drive the formation of cell protrusions, and modulate microdomain organisation. Moreover, cortex-associated myosin motors can generate contractile forces, effect transport along actin fibres and modulate cortical tension and cell rigidity [66,67]. PM receptors can establish dynamic contacts with the cortex, often involving linker proteins that simultaneously bind actin and the cytoplasmic domains of PM proteins (Table 1) [68]. Receptor-cytoskeleton anchoring directly impacts on receptor mobility and influences receptor function [69].

**Table 1:** Summary of proteins that interact with HIV receptors and their role in virus entry. Abbreviations: KD: knockdown; VS: virological synapses; OE: overexpression.

Protein	Description	Evidence related to role in HIV entry	Refs
Lck	Tyrosine kinase. Can bind cytoplasmic domain of CD4. Could mediate functional CD4-actin links [[80],[81],[80–82]]. Signals in T-cell immunological synapses.	Lck participates in the activation of filamin-A, leading to enhanced F-actin cross-linking.	[78]

ERM proteins (ezrin, radixin, moesin)	Actin-binding proteins [84,85]. Can bind PM components and cortical actin in active conformation. Folded when inactive.	Moesin and ezrin activate upon CD4 binding by soluble gp120 or HIV particles. Moesin regulates actin reorganisation that drives Env-induced CD4/CXCR4 redistribution and clustering on human T cells. Moesin KD inhibits HIV particle entry/infection and fusion with Env-expressing cells.	[86]
		ERM proteins regulate HIV infection. KD experiments with TE671, 293T, HeLa cell lines expressing HIV receptors and co-receptors.	[87]

Filamin-A	Adaptor and actin-binding protein.	Filamin-A redistributes to sites of CD4 engagement (by anti-CD4 Ab or by gp120) in human Jurkat T cells. Molecular structure modelling suggests CD4 could be bound by Filamin-A and Lck simultaneously. Filamin-A interacts with CCR5/CXCR4 and their signalling pathways. Implicated in Env-induced CD4 clustering and F-actin rearrangements. Mediates activation of RhoA-ROCK-LIMK-cofilin signalling pathway upon CD4 binding, which results in cofilin inactivation.	[88]

ITK	Inducible T-cell Kinase (Tec family of tyrosine kinases) [[89], [90], [91]].	Mediates Env-induced actin rearrangements and signalling required for HIV entry downstream from chemokine co-receptors. Inhibition of ITK in human CD4^+^ CXCR4^+^ Jurkat T cells partially blocks viral entry.	[92]

Rho-family GTPases	Activated by chemokine ligand binding of CCR5/CXCR4, involved in actin rearrangements for chemotaxis.	Rac-1 GTPase is activated upon CCR5/CXCR4 engagement by HIV Env, stimulating F-actin reorganisation and aiding Env-induced cell-to-cell fusion.	[93]
		The activity of Rho GTPases (e.g., RhoA) leads to re-arrangements required for membrane fusion. This activity is regulated by ERM proteins.	[94]

Cofilin	Actin depolimerising factor; inactive in resting T cells, active in activated T cells.	In cells with active cofilin, HIV Env binding to CD4 transiently activates LIMK-1. This inactivates cofilin, blocking CXCR4 internalisation and promoting cortex reorganisations for HIV receptor/co-receptor clustering. Dense barrier of F-actin and proteins forms at Env contact sites. Subsequent HIV-Env induced signalling through CXCR4 transiently activates cofilin removing the cortical barrier at the instance of virus entry.	[20,95]

Gelsolin	Actin-binding protein.	Regulates actin levels and dynamics during early HIV infection in CD4^+^ T cells. Gelsolin KD and OE inhibit Env-induced receptor/co-receptor redistribution and clustering, impairing viral fusion and infection.	[96]

Syntenin-1	PDZ adaptor protein. Can mediate links between PM receptors and the cytoskeleton.	Regulates Env-induced actin polimerisation and PIP_2_ lipid accumulation at sites of contact with virus; inhibits viral entry. Co-localises with CD4 at Env contact sites in PM of CD4^+^ T cells (CEM-T4). Associates with CD4 cytoplasmic domains, possibly interfering with Lck-CD4 binding.	[97]

Drebrin	Actin-binding protein.	Regulates HIV-induced actin polymerisation for viral entry. Can bind F-actin and CXCR4 cytoplasmic domain. Accumulates at Env contact sites. Has inhibitory effect on HIV entry and Env-mediated cell-cell fusion.	[98]

Evidence suggests that various actin-binding and adaptor proteins (e.g. Ezrin/Radixin/Moesin [ERM] proteins, filamin-A, gelsolin, syntenin-1, drebrin) can establish links between HIV receptors and F-actin (Section 4.5). Additionally, p56Lck (Lck), an intracellular, lymphocyte-specific, Src-family tyrosine kinase, directly binds the cytoplasmic domain of CD4. This binding confers signalling potential to CD4 and also inhibits CD4 internalisation [[70], [71], [72]] leading to very low CD4 endocytosis rates in Lck-positive cells (~0.2–0.8%/min; compared to 10-fold higher rates in Lck-negative cells) and low steady state levels of intracellular CD4 [71,73]. Lck is known to play essential signalling roles in T-cell activation in immunological synapses [[74], [75], [76]] and in virological synapse-mediated HIV cell-to-cell transmission [77]. Moreover, in in vitro experiments, Lck can participate in the activation of filamin-A, leading to enhanced F-actin cross-linking [78], and the formation of artificial virological synapses [79]. Although not essential for HIV infection (HIV can infect Lck-negative cells such as macrophages), Lck association with CD4, and possible physical and functional links to the cortex [[80], [81], [82]], may influence the diffusive properties of CD4 [73] and impact on virus engagement and entry kinetics in T cells. Similarly, for the GPCR CCR5, cytoplasmic domain interactions with G proteins may influence receptor conformation and HIV engagement [83].

In the Kusumi “picket-fence” model of PM organisation [99], cortical actin filaments can form 40–300 nm corrals consisting of actin “fences” and transmembrane protein “pickets” anchored to the actin fences [e.g. CD44 [69]] [61]. These corrals restrict the long-range diffusion of membrane proteins via the presence of immobilised pickets and via the presentation of fences encountered by the cytoplasmic domains of membrane-associated proteins [61,64]. In this model, proteins can be transiently confined within a corral for short periods, generally in the order of tens of milliseconds: Within the corral, proteins can diffuse randomly and rapidly (diffusion coefficients ca. 5 μm^2^/s) [61]. Longer-range diffusion takes place at much slower rates (ca. 0.2 μm^2^/s on average) and is dominated by hop-diffusion, i.e. relatively rare events in which molecules ‘hop’ across boundaries into adjacent corrals [61]. The diffusion within a corral can only be detected with imaging techniques capable of high temporal resolution (10 μs–1 ms), and spatial localisation precision less than the dimensions of the actin corrals. In this model, oligomerisation and clustering of membrane proteins leads to a dramatic reduction in their hop rate and long-range diffusion, compared to that of monomeric proteins. This reduction is much greater than expected if diffusion was due solely to Brownian motion [61]. ‘Corralling’ suggests a mechanism for the clustering and oligomerisation of signalling receptors that may facilitate signal-transduction and other functions [61,64,100]. In the context of virus entry, ‘corralling’ may enhance the co-clustering of receptors and co-receptors during viral engagement (Section 4.4).

The diffusion modes discussed above exhibit different temperature dependencies. For short-range random diffusion within corrals, the diffusion coefficient (*D*) in a given dimension increases linearly with temperature (*T*) as *D* = *k*_*B*_*T*/*γ*, as described by Einstein's Brownian motion theory. Here, *k*_*B*_ is Boltzmann's constant and *γ* is the friction coefficient for proteins in a lipid bilayer, which is proportional to membrane viscosity and protein size. By contrast, longer-range, hop-diffusion is an energy-driven process that follows an Arrhenius model (stochastic escape from an energy barrier *E*_b_), with the diffusion rate depending exponentially on the reciprocal of the temperature (*D* ∝ *e*^−*E*_b_/*k*_B_*T*^) [101]. This is important when considering data from experiments carried out at different temperatures.

## 2 Measurement techniques

### 2.1 Receptor-ligand binding assays and force-spectroscopy techniques

Virus entry is generally thought to require multiple Env-receptor/co-receptor interactions. Knowledge of how binding occurs is essential to fully understand virus entry and may aid the design of novel antiviral strategies.

A receptor-ligand binding reaction is characterised by its equilibrium dissociation constant, *K*_d_ = *k*_off_/*k*_on_, where *k*_off_ is the off-rate constant (s^−1^) and *k*_on_ is the on-rate constant (M^−1^s^−1^). At chemical equilibrium, a steady state is reached in which both bound and unbound receptor concentrations are constant in time, with the on-rate for receptor-ligand association, *k*_on_[*R*][*L*] (M/s), being equal to the off-rate for dissociation, *k*_off_[*RL*] (M/s). Here, [*L*], [*R*] and [*RL*] are the concentrations of ligand and unbound and bound receptors, respectively. *K*_d_ (in units of concentration, M) corresponds to the equilibrium concentration of ligand for which the receptor binding sites are half occupied. It is often used to quantify the strength or affinity of a receptor-ligand interaction, with lower *K*_d_ values indicating higher binding affinities. The inverse of *K*_d_, known as the equilibrium association constant, *K*_a_ (M^−1^), is also often used. While useful to compare various ligand molecules in the same conditions, affinity alone does not provide the full picture. For instance, a receptor could have the same affinity for two different ligands, but one ligand-receptor pair could have both a high binding rate constant and high dissociation rate constant, while the other ligand-receptor pair could have both low binding and dissociation rate constants, leading to very different binding kinetics and requirements for effective engagement in the context of multivalent interactions such as those involved in virus entry. Avidity is therefore important: for the typically short-lived molecular bonds of HIV gp120 with individual receptor and co-receptor molecules (Section 4.1), multiple bonds (aided by receptor clustering, Env's trimeric nature and Env clustering) will be required to extend the overall bond lifetime in order to sustain the interactions that trigger cellular signalling and virus entry.

While it is preferable to have information on both the *k*_on_ and *k*_off_ to characterise binding interactions, a binding rate in vivo depends on a number of complex physiological conditions that are difficult to measure, reproduce and control experimentally. For receptor-virus binding, these include the number and mobility of viral particles, the proximity of viruses to target cells, the flow conditions (in blood and tissues), the density, clustering and mobility of receptors on the surface of target cells, and whether it is cell-free or cell-to-cell transmission. For instance, the binding rate of HIV Env to its receptors/co-receptors is most likely faster in virological synapses, as there is a high probability of re-binding due to virion confinement. Nevertheless, experiments with cells or with surface-immobilised molecules in vitro can be used to gain some insights into Env/virus binding, including characterising the dissociation rate of relevant bonds following virus engagement. Thus, techniques such as single molecule force spectroscopy can interrogate interactions in living cells in near-physiological conditions by measuring bond lifetime (*τ* = 1/*k*_off_) and strength (bond-rupture forces).

Traditionally, various types of biochemical receptor-ligand binding assays have been used to measure *K*_d_, *k*_on_ and *k*_off_. Early assays employed isothermal titration calorimetry (ITC) and radioactive methods, with more recent methods including Förster resonance energy transfer (FRET), flow cytometry [e.g. fluorescence-activated cell sorting (FACS)], fluorescence correlation spectroscopy (FCS) and surface plasmon resonance (SPR) [102]. The equilibrium constant *K*_d_ can be measured via equilibrium assays (*k*_on_ and *k*_off_ are hence not measured), whereas kinetic assays are more informative and measure both *k*_on_ and *k*_off_ from which *K*_d_ can be obtained [103]. In particular, ITC consists of equilibrium assays for the direct determination of the thermodynamic parameters (*K*_a_in the range mM to nM, Gibbs free energy of association, enthalpy and entropy changes, stoichiometry) of ligand-binding reactions in equilibrium, with both binding partners in solution [104,105]. SPR biosensors [106] have high sensitivity and can readily perform kinetic binding assays with one binding partner typically attached to a surface and the other in solution. All these assays measure ensemble averages and, therefore, transient events and differentiated behaviours within a heterogeneous mixture are typically not detected (SPR can actually detect some heterogeneity in either binding partner (not both) as well as transient events in the form of conformational changes). With few exceptions [[107], [108], [109]], the majority of ensemble kinetic assays have been performed in vitro and not on cells and the results may not be representative of receptor-ligand interactions in living cells. The ensemble average binding and dissociation rates measured for the equilibrium state of a binding reaction in 3D solution or for surface-immobilised molecules will be different from those that apply to the non-equilibrium binding interactions near the PM of a living cell. For instance, measurements from ensemble assays in 3D solution depend on protein concentration and diffusion rate in 3D, while measurements with surface-immobilised proteins preclude diffusion of one binding partner. In both cases, binding and dissociation take place in conditions very different to those encountered by viruses near the surface of a cell, resulting in differences in binding affinities [110].

As an alternative, and in the context of virus entry into living cells, single molecule force spectroscopy offers advantages in that it can directly probe and characterise individual macromolecular binding interactions. For example, single molecule sensitivity enables the measurement of binding events one bond at a time, as opposed to ensemble-average behaviour; dynamic binding changes can be monitored with high temporal resolution, and versatile force pulling/pushing experiments in living cells can provide useful information in physiological conditions. Experiments in living cells are particularly suitable for transmembrane proteins, such as the HIV receptors, that require a lipid environment to maintain their native structure [111]. Force spectroscopy techniques typically detect forces at the picoNewton level (the relevant scale for macromolecular interactions) with sub-nanometre spatial precision and millisecond (or better) temporal resolution. The most widely employed techniques are atomic force microscopy (AFM; aka molecular force probes), optical tweezers (OT) and magnetic tweezers (MT) [112]. Biomembrane force probes (BFP) in micropipette assays can also be used [[113], [114], [115], [116]]. These techniques use a probe [e.g. a flexible AFM cantilever tip, bead (OT, MT) or deformable cell/bead (BFP)] to apply pulling or pushing forces on single molecules in in-vitro assays or on the surface of living cells. In a calibrated setup, forces can be accurately measured from the probe deformation (AFM, BFP) or from the probe (bead) position (OT, MT).

**Fig. 4:**
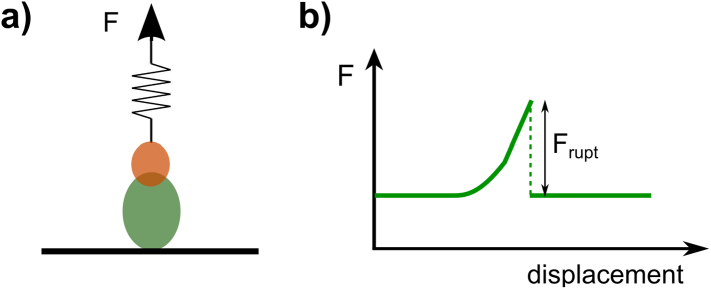
Force spectroscopy to probe single molecule bonds. a) Schematic of force (F) application to pull a macromolecular bond. b) Qualitative force versus displacement curve as the bond is pulled at constant pulling speed and force loading rate R. The force required to break the bond is F_rupt_.

To investigate single molecular bonds, probes are typically functionalised [e.g. polystyrene beads coupled to a monoclonal antibody (mAb)] to facilitate attachment to specific molecules on a cell or glass surface and then pulled away from the attachment point (Fig. 4a). Experiments can be carried out by applying a constant force to measure bond lifetimes, or pulling at constant speed to obtain force-versus-displacement curves that yield characteristic bond-rupture forces (dynamic force spectroscopy, Fig. 4b). The interpretation of force spectroscopy experiments is based on the idea that bond dissociation in a viscous fluid can be described as the escape of a thermally-fluctuating Brownian particle from a transiently confining potential energy well [theory by Kramers [117], Bell [118], Evans [[119], [120], [121], [122]] and Dudko [[123], [124], [125]]]. In the absence of force, molecular bonds have an unstressed lifetime (*τ*_0_) related to the probability that thermal fluctuations (collisions with the surrounding fluid molecules) provide enough energy to overcome all the potential barriers leading to bond dissociation. Under force application, such potential barriers are lowered, increasing the probability of bond rupture and decreasing bond lifetime. By carrying out experiments at different forces and different pulling rates (force loading rates), *τ*_0_ can be measured and a bond's unstressed dissociation rate,*k*_off, 0_ = 1/*τ*_0_ obtained.

When pulling cell-surface receptors in living cells, it is important to consider the stiffness of the actin cortex (that typically shows an elastic response with a linear force-versus-displacement trace) and the viscoplastic mechanical properties of the membrane (typically measured as a non-linear force versus displacement), in addition to the actual bond of interest. In fact, these different contributions and their signatures can be exploited to measure receptor-cytoskeleton attachments, as previously demonstrated for cell-adhesion receptors (e.g. integrins, cadherins) [[126], [127], [128]].

Force spectroscopy can also be used to improve our limited knowledge of the forces and local mechanical properties relevant to the virus-cell interactions leading to virus entry. For instance, local changes in membrane tension [129] and bending rigidity [130,131] can be measured and the influence of molecular conformation and flexibility can be studied through molecular stretching [132] and protein unfolding [133] experiments.

Additionally, OT can be used to trap and manipulate entire cells [134] or virions [135] in 3D culture fluid with high flexibility, enabling controlled positioning for force sensing experiments. This has allowed, for example, the study of HIV transfer in live lymphocyte virological synapses within sealed (safe) chambers [134]. A method for tethering an individual HIV particle via a DNA tether to an optically trapped bead has also recently been reported as a promising technique for studying virus-cell interactions [136]. BFP also offers versatile positioning capabilities and is applicable to force sensing at cell-cell interfaces in suspension. More recently, novel single-molecule force-mapping tools have been presented, based on extensible linkers [polyethylene glycol (PEG), single stranded DNA or DNA hairpins] and FRET pairs of molecules (donor/acceptor or emitter/quencher pairs) as reporters of linker extension or rupture [[137], [138], [139], [140], [141], [142]]. These sensors can generate mechanical traction force maps in living cells (incl. synapses) with a sensitivity of tens of pN.

### 2.2 Imaging methods

The properties of cell-surface receptors have typically been studied using diffraction-limited fluorescence microscopy in conjunction with directly- or indirectly-linked fluorescent reporters. Selected proteins can be tagged with fluorescent labels (fluorophores) of various excitation/emission wavelengths and brightness levels. The most common fluorophores are small organic dyes (<1 kDa, <1 nm size), fluorescent proteins (FPs; e.g. green fluorescent protein [GFP] and its derivatives, ~27 kDa, ~3–5 nm size) and inorganic semiconducting quantum dots with size-dependent fluorescence properties [143,144]. The most widespread techniques for following specific proteins are immunolabelling and genetic tagging [143]. In immunolabelling, specific antibodies (Ab; often a mAb) are used to label molecules of interest, with the Ab being conjugated to a fluorophore directly or via a secondary Ab. This method is particularly convenient for transmembrane proteins, such as viral receptors, that are accessible on the cell surface. However, the valency and size of Ab molecules (150kD; 10–20 nm for a divalent IgG) can induce cross-linking and other artefacts that influence the biophysical properties of membrane proteins [143]. The use of monovalent Ab fragments (FAbs) or nanobodies (single-domain Abs), may reduce these problems but is generally associated with loss of avidity [145,146]. An alternative is to use genetic tagging to fuse a FP [or other reactive protein domain, e.g. SNAP [147,148]] to a protein of interest. The genes encoding these fusion proteins must then be transfected into cells for either transient or stable expression. Although very useful, FPs and similar proteins can also be problematic through aggregation of FP tags, interference with normal protein-protein interactions and function, and off-target effects if expression levels are not carefully controlled (though techniques such as CRISPR/CAS9 can be used to ensure more physiological levels of expression). Small genetically encoded peptide tags can be used to avoid some of the problems associated with larger protein domains [143,149]; for example FlAsH/ReAsH have been used to label virus particles engineered to contain tetra-cysteine tags [150,151], though these dyes often show high backgrounds when used in cells. Quantum dots must be linked to their targets through specific Abs or ligands, and also have the potential to cross-link.

Fluorescence recovery after photobleaching (FRAP) has frequently been used to study fluorescently-labelled protein populations on living cells [144,152]. More recently, single molecule fluorescence imaging together with single particle tracking (SPT) [153,154] has enabled the localisation of diffraction-limited fluorescent spots with nm spatial resolution (for sparse enough spots), and ms (or better) temporal resolution, in living cells, in real-time. SPT can be used to measure protein diffusion [155,156] and to determine the numbers of molecules that move together in oligomers or clusters (through analysis of intensity levels and step-wise photobleaching decay of the emission from the fluorescently labelled molecules) [157,158]. Fluorescence correlation spectroscopy (FCS) is also a well-established technique to measure protein mobility and concentration [159,160]. FRET [144,159] can be used to measure protein interactions and proximity and has been applied to the real-time detection of virus fusion in living cells [161]. As with FRAP, both FRET and FCS measure protein populations, in contrast to SPT methods. It is important to be aware that cell contact with glass surfaces can induce receptor clustering, mobility changes [162,163] and cytoskeletal re-arrangements [164], thus methods that involve imaging glass-membrane interfaces, such as total internal reflection fluorescence microscopy (TIRFM), should be interpreted with care.

The labelling of HIV particles has been possible for over a decade, by tagging either viral proteins, such as Gag, Vpu and Env, and/or by incorporating fluorescent membrane lipids. This has enabled, for instance, measurements of the mobility of single virions on cells [[165], [166], [167]], studies of Env and Env conformational states on virions [168,36], visualisation of virus transfer across virological synapses [169,134], virus-to-cell fusion [170], and the intracellular behaviour of HIV particles and sub-viral components within living cells [151,[171], [172], [173]].

Given that the sizes of HIV receptors, HIV particles and HIV-Env are below the diffraction limit of conventional optical fluorescence microscopy at visible wavelengths (~200–300 nm), the use of super-resolution imaging (SRI) is strongly desirable. SRI methods achieve sub-diffraction limit resolutions down to ~10–100 nm laterally and ~10–300 nm axially, depending on the technique [174]. To date, however, the application of SRI to cell-surface proteins in general, and HIV receptors in particular, has been limited [though SRI has been used to study the distribution of Env on HIV particles [168] and stoichiometry in pre-fusion HIV entry complexes [175]]. SRI methods include stimulated emission depletion microscopy (STED), photo-activated localization microscopy (PALM), stochastic optical reconstruction microscopy (STORM) and structured-illumination microscopy (SIM) [174,[176], [177], [178], [179], [180], [181]]. While SRI initially suffered from a low temporal resolution and was limited to fixed cells, recent advances in single-point scanning and illumination/scanning parallelisation techniques, reduced light dosage strategies, combinations of multiple techniques and advances in reconstruction algorithms, have enabled the application of SRI to living cells [[181], [182], [183], [184], [185]]. Additionally, STED-FCS has enabled the analysis of live cell PM lipid (and protein) mobility with simultaneous high spatial and high temporal resolution [186].

Electron microscopy (EM) has been used to visualise HIV receptors and entry events at high spatial resolution (sub-nm), often in conjunction with immuno‑gold labelling [187,188]. However, currently EM can only be used with fixed material and dynamic information is lost. AFM, which can achieve nm spatial resolution, has also been used to visualise aspects of HIV fusion with model membranes [189] and the organisation of isolated virions and cell-associated virus structures on infected lymphocytes [2,190]. AFM has the advantage of being suitable for imaging biomolecules in liquid with relatively simple sample preparation and non-destructive imaging on living cells can be achieved. As a scanning-probe technique, AFM can suffer from slow image-acquisition times (typically of the order of seconds), though recent high-speed AFM has achieved image times of ~ 10–20 ms for full proteins [112,191,192].

## 3 Properties of HIV cell-surface receptors

### 3.1 Numbers of receptor molecules in the plasma membrane

Table 2 shows examples of the numbers of HIV receptor molecules (CD4, CCR5 and CXCR4) measured on various cell types [193]. Most of these measurements were derived from flow cytometry studies. The data show that receptor numbers on T-cell lines commonly used to propagate HIV vary considerably [194] and that these can be substantially different to those found on primary cells. Crucially, these studies do not usually give information of receptor density, i.e. the number of receptors per unit area of membrane, a key determinant of the probability that the molecular encounters required for HIV entry will occur. Primary cells, such as peripheral blood lymphocytes from healthy donors, tend to show less variability in receptor numbers, though there is some donor variation. In particular, human primary CD4^+^ T cells have 15,000–25,000 CD4 molecules/cell and 2000–7000 CCR5 molecules/cell [195]. A number of stably transfected and cloned cell lines have been produced that express variable numbers of receptor molecules with some, such as HeLa-TZM-bl [196,197], used extensively as reproducible HIV reporter lines. In the inducible ‘Affinophil’ HEK293 cell line, receptor properties can be studied over a range of CD4: CCR5 ratios and expression levels [198].

**Table 2:**
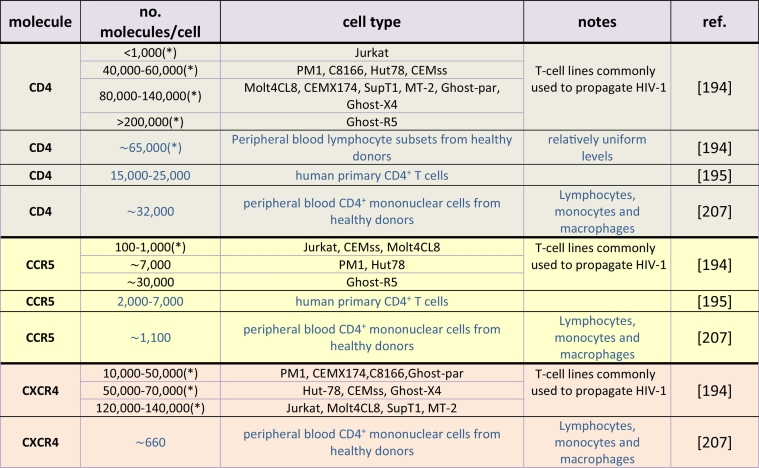
Measured average numbers of HIV receptor molecules in different T-cell lines (black) and primary cells (blue). (*): Numbers are ABS/cell (antibody binding sites per cell) as opposed to molecules/cell. Data from [194,195,207].

Cell surface expression of viral receptors/co-receptors is dynamic and can be modulated in time: CCR5 and CXCR4 can be internalised [199,200], following binding of a cognate chemokine agonist or through cross-signalling and/or hetero-oligomerisation [201,202], and subsequently degraded or recycled to the PM [203]. CD4 molecules can also be endocytosed at rates that are influenced by Lck and protein kinase C (PKC)-mediated phosphorylation [71]. Additionally, significant variations in expression levels can occur over time and may be associated with cell maturation or activation state [194]. Receptor levels also change on primary cells from HIV infected patients, e.g. peripheral blood CD4^+^ T cells have been reported to express ~46,000 CD4 molecules/cell [204] and ~4000–24,000 CCR5 molecules/cell [205], i.e. similar levels of CD4 but increased CCR5 expression compared to cells from healthy donors (Table 2). How exactly these variations in receptor number impact on virus transmission in vivo and subsequent pathogenesis remains unclear, though the increase in CCR5 expression on peripheral blood CD4^+^ T cells from infected patients correlates with viral load [205] and CCR5 expression on primary CD4^+^ T cells from healthy donors also positively correlates with R5 HIV replication [195].

CCR5 levels on CD4^+^ human osteosarcoma cells (HOS) have been found to influence the efficiency of R5 HIV entry and virus production, with a 7-fold increase in CCR5 expression increasing virus entry 2–3 fold and increasing viral production 30–80-fold after a single replication cycle [206]. Evidence of the interdependency of CD4 and CCR5 expression on HIV infection has been provided by experiments with CD4^+^ HeLa cells transfected with CCR5. In cells with high CD4 levels (~450,000 molecules/cell), efficient infection by macrophage-tropic HIV was supported even for low CCR5 levels (700–2000 molecules/cell). However, in cells with low CD4 levels (~10,000 molecules/cell), a threshold CCR5 level of 10,000–20,000 molecules/cell was required for efficient infection [196].

We estimate that a typical T-cell may have 1–2 CD4 molecules and ~0.2 CCR5 molecules per actin corral [assuming an average corral size of ~200 nm [61,64] and average uniform receptor densities of 25,000 CD4 molecules and 3000 CCR5 molecules per cell, no aggregation and, as an approximation, oblate ellipsoidal cell shapes approx. 20 μm in diameter]. Although approximations, these estimates suggest that slow, longer range, hop-diffusion between corrals, or signalling-induced perturbation of corrals is likely to be necessary to ensure virus particles encounter the requisite number of receptor/co-receptor molecules to initiate fusion.

### 3.2 Mobility of HIV receptors

The measurements for the lateral mobility of CD4 and CCR5 published to date are summarised in Table 3, Table 4, respectively. We have not found any data for CXCR4. The tables contain measurements at 20 °C and 37 °C; hence the temperature dependence of the different diffusion coefficients explained in Section 1.3 needs to be considered. With the exception of one SPT study, all data were derived from FRAP experiments.

**Table 3:**
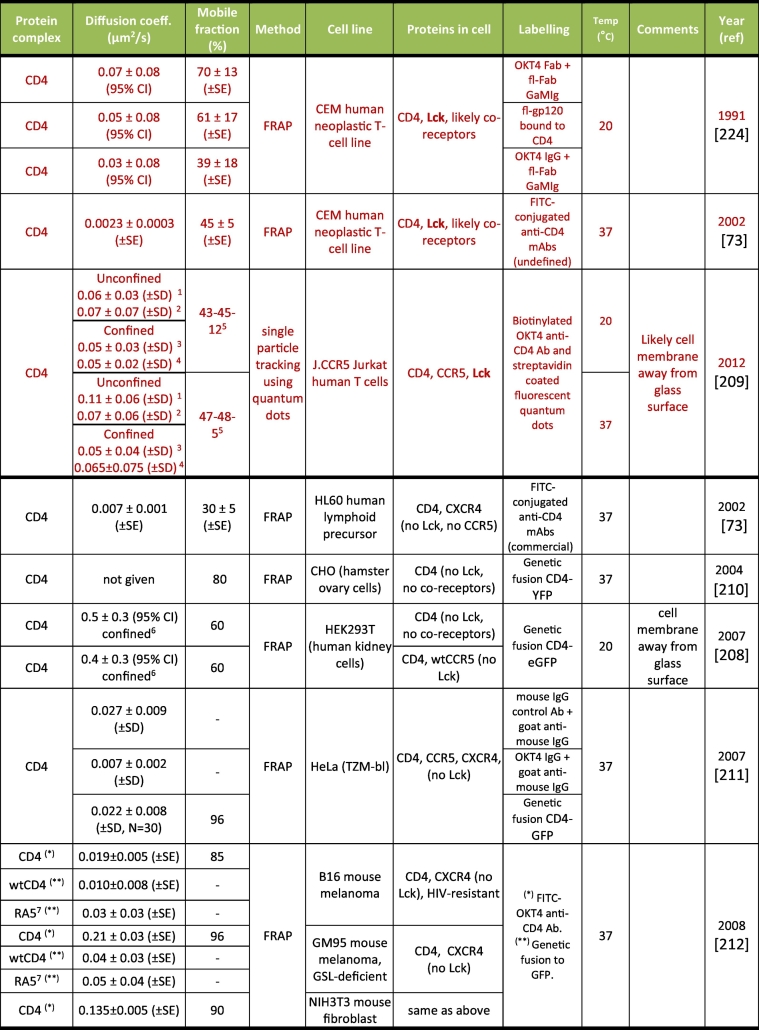
Summary of diffusion measurements for cell-surface receptor CD4. Data highlighted in red at the top correspond to Lck^+^ T cells, while data at the bottom correspond to Lck^−^ cells. SE = Standard Error of the mean; CI = Confidence Interval of measurement; A 95% CI is equivalent to ±1.96 SD; SD = Standard Deviation; N = number of independent data points measured, with SE = SD/$\sqrt{N}$; GSL = glycosphingolipids; QD = quantum dots. ^1^For unconfined receptors. ^2^For unconfined segments of transiently confined receptors. ^3^For confined receptors. ^4^For confined segments of transiently confined receptors. ^5^Percentages of receptors showing unconfined diffusion, transiently confined diffusion and permanently confined diffusion, respectively. ^6^A single population displaying confined diffusion was observed in cells with and without CCR5. ^7^CD4 mutant that localises to non-raft membrane micro-domains. For most studies except those with explicit comments, it was unclear whether the glass-attached or unattached membrane was imaged.

**Table 4:**
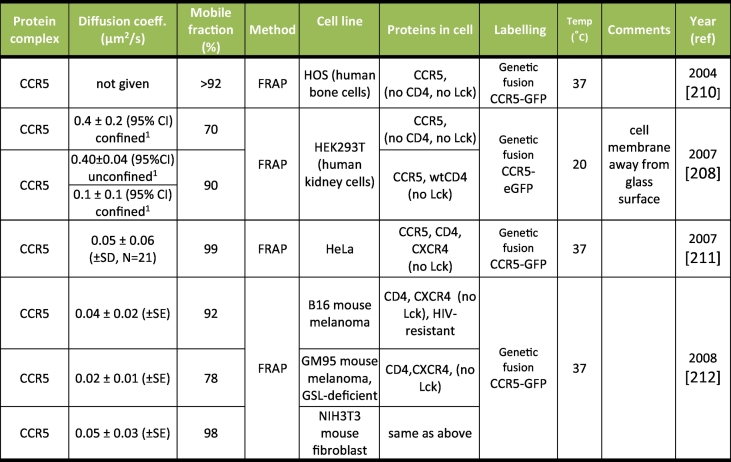
Summary of diffusion measurements for cell-surface receptor CCR5. SE = Standard Error of the mean; CI = Confidence Interval of measurement; A 95% CI is equivalent to ±1.96 SD; SD = standard deviation; N = number of independent data points measured, with SE = SD/$\sqrt{N}$; GSL = glycosphingolipids. ^1^A single population displaying confined diffusion was observed in cells with no CD4, and two populations displaying confined and unconfined diffusion were measured in the presence of CD4. For most studies except those with explicit comments, it was unclear whether the glass-attached or unattached membrane was imaged.

Overall, the results for CD4 mobility (Table 3) show considerable variation. The majority of measurements suggests a lateral mobility of ~0.05 μm^2^/s (see data for Lck^+^ T cells at 20 °C), but results an order of magnitude above and below this number, and uncertainty as to the impact of Lck association, indicate that additional, carefully controlled analyses, under well-defined experimental conditions, are necessary.

Two studies have reported the existence of sub-populations of receptors and co-receptors displaying distinct diffusive modes [208,209]. SPT of CD4 revealed sub-populations with unconfined, transiently confined and permanently confined behaviours on the surface of Jurkat T cells [209]. Unconfined diffusion corresponds to random Brownian motion with a mean square displacement [*MSD*(*τ*) = 〈[*x*(*t* + *τ*) − *x*(*t*)]^2^〉, where *x* is the position, *t* is the time, *τ* is the time lag and the angle brackets indicate an average over all *t* values in a measured diffusion track] that depends linearly on the time lag *τ*. For confined diffusion, the *MSD* deviates from this linear behaviour and saturates at long lag times, indicating trapping in a confined region [155]. Approx. 40–50% of all CD4 molecules tracked displayed unconfined diffusion, ~40–50% showed transiently confined diffusion and 5–10% displayed permanently confined diffusion. The diameters of the confinement areas were ~200 nm [209]. This is partially consistent with the hop-diffusion model, though the measured diffusion coefficients were lower than expected for individual receptor molecules. It is possible that the confined and unconfined fractions correspond, respectively, to CD4 associated to, or free of, Lck. Alternatively, the different diffusion modes could correspond to different receptor aggregation or conformational states. Interestingly, diffusion constants measured away from the glass surface were significantly higher than most other measurements and closer to the coefficients expected for long-range diffusion across cortical boundaries within Kusumi's “picket-fence” model (Section 1.3).

The presence of actin-binding proteins filamin-A, syntenin-1, drebrin and ERM proteins (Section 4.5) can anchor HIV receptors to the actin cytoskeleton, and in principle can lead to reduced receptor mobility, possibly stabilising/enhancing the molecular interactions necessary for virus entry (i.e. virus binding and receptor clustering). On the other hand, directed motion of anchored receptors to virus attachment sites via active cytoskeleton rearrangements, may also favour virus binding. New experiments to measure whether such links affect receptor/co-receptor mobility and hinder or promote virus entry would be extremely interesting. Additionally, robust characterisation of the diffusive mobility of HIV receptors and co-receptors before and after virus engagement will help towards a better understanding of virus entry dynamics.

Alterations in PM composition such as cholesterol depletion [210], sphingomyelinase treatment [211] or glycosphingolipid removal [212] also possibly affect receptor distribution and mobility. CD4 is palmitoylated, a modification that is believed to target the protein to lipid raft domains [213]. The structural integrity and function of CCR5 and CXCR4 also seem to require PM cholesterol [199,214,215]. Thus, perturbing PM lipid composition may influence the properties of both proteins. More experiments are needed to understand the relevance of these lipid-protein interactions in the context of virus entry [62,[216], [217], [218], [219], [220], [221], [222]], especially since recent evidence has suggested that HIV fusion occurs at the interfaces between liquid ordered and liquid disordered PM microdomains [223].

As for CD4, all CCR5 measurements reported to date (Table 4) have used transfected non-lymphoid cells and the majority suggests a lateral mobility of about 0.04 μm^2^/s, with coefficients differing by an order of magnitude or more overall. In addition, all studies used FP-tagged CCR5 proteins: our own studies with GFP-tagged chemokine receptors indicate that the kinetics of endocytic trafficking are influenced by FP tags (unpublished observations).

For both CD4 and CCR5, the observed large variability in the published data likely arises from a combination of the low precision of some of the methods used, different labelling methods and assay temperatures, together with natural variations between cell lines and cell types. For instance, many of the measurements have relative errors close to 100%, making it difficult to extract statistically significant differences and meaningful comparisons between experiments. As PM contact with glass can modify receptor mobility [162,163] or induce receptor clustering and cytoskeletal reorganisation [164], measurements of receptor mobility in the PM away from glass is necessary.

Additionally, as clustering of receptors by crosslinking with multivalent Abs can modify receptor diffusion [224,211], the use of small tags that do not alter protein mobility or interfere with key cytoplasmic domains of the receptors (e.g. those that may mediate anchoring to the cytoskeleton) are preferred. The nanoscale topology of the membrane (e.g. microvilli) can also affect the interpretation of mobility measurements. As typically lateral (2D) diffusion is measured, vertical movements up and down membrane features can be interpreted as stalling, affecting the measured diffusion coefficients. Complementary high-resolution topography explorations using EM, AFM or scanning ion conductance microscopy would be of added benefit [225].

### 3.3 Oligomerisation and conformational variation of receptors in the plasma membrane

The states of aggregation or oligomerisation of viral receptors are also likely to influence their mobility, particularly when considering hop-diffusion, HIV entry and leukocyte function. Signalling pathways downstream from receptor engagement, and receptor-cytoskeleton links, may also vary for different receptor oligomerisation states. However, only a few studies have considered these effects to date.

Experimental evidence for CD4 oligomerisation, and theoretical oligomeric structures, was reviewed in 1998 [226]. Several studies have reported the existence of both monomeric and dimeric CD4 in different cell lines, and specifically on the surface of immune cells [[227], [228], [229]]. Moreover, a number of studies have suggested that CD4 dimers preferentially function as immune co-receptors for T-cell signalling [[230], [231], [232]], whereas it is unclear whether the preferred form for HIV binding/entry is monomeric or dimeric [229,233]. The dimeric association, through the D4 Ig domain, was seen in a crystal structure of soluble CD4 [230] and D4-mediated CD4 dimerisation on the surface of T cells was subsequently shown to be essential for CD4 immune function [231]. Predominantly covalent CD4 dimers have also been reported in Langerhans' cells (a type of HIV-sensitive dendritic cell in the skin) [233]. Covalent CD4 dimers formed through disulphide links between D2 domains have also been reported [229,232], with dimer formation possibly linked to conformational changes in CD4. A role for Lck in disrupting possible dimeric interactions mediated by the CD4 intracellular domains has been proposed, arguing that CD4 is mostly bound to Lck and monomeric in the PM of human T cell lines [162]. However, HIV fusion and entry experiments have suggested that eliminating CD4 D2 disulphide bonds, which prevented the formation of D2-linked CD4 dimers, led to a 2–4 fold increase in viral entry, suggesting that monomeric CD4 may enable more efficient HIV infection [229].

As for the HIV co-receptors, CCR5 and CXCR4 are GPCRs, for which homo- and hetero-dimerisation have been a focus of numerous studies [201]. The presence of CXCR4 and CCR5 homodimers, and heterodimers of CCR5 and CXCR4 with other chemokine receptors or GPCRs has been reported [202,[234], [235], [236], [237], [238], [239], [240]]. Most of these studies demonstrate the presence of pre-formed, constitutive dimers in the PM, independent of ligand addition [chemokines are known to induce dimerisation, clustering and redistribution of chemokine receptors in the PM [199]]. However, there is evidence that CCR5 monomers are likely to mediate HIV infection based on experiments showing that HIV replication was blocked in human peripheral blood mononuclear cells (PBMCs) ex vivo, and in vivo in mouse models, by CCR5 dimerisation: this dimerisation, similar to that induced by RANTES (a natural chemokine with anti-HIV activity) was induced by anti-CCR5 mAbs that did not interfere with HIV gp120 binding or chemokine binding, and that did not trigger CCR5 signalling or internalisation [241]. The same authors reported that co-expression of CCR5 with CD4 and CXCR4 in the PM of HEK 293T cells and CD4^+^ CXCR4^+^ T cells was able to disrupt binding, entry and infection by X4-tropic HIV through the formation of CD4-CCR5-CXCR4 hetero-trimers that altered the conformation of existing CXCR4 homodimers and CXCR4-CD4 heterodimers [242]. Receptor/co-receptor oligomerisation reduces long-range hop diffusion (consistent with Kusumi's “picket-fence” model) and impairs receptor/co-receptor recruitment for virus entry, and fits with observations of reduced HIV fusion when CD4-diffusion is restricted [211].

The different oligomerisation states of receptor molecules may be linked to the reported CCR5 conformational heterogeneity [[243], [244], [245]] that suggests that distinct CCR5 conformational states can influence the distribution and abundance of CCR5 in the PM, and can have different ligand binding affinities and down-regulation rates [243]. Accordingly, different CCR5 conformations might also influence HIV binding and entry [244,245]. CXCR4 molecules have been found to be highly heterogeneous on different cells, both in terms of their structure and function, with different CXCR4 isoforms possibly being responsible for HIV entry and/or chemotactic responses [246].

These data notwithstanding, differences in receptor expression levels, relative densities, the mean distance between receptor molecules, diffusion properties and recruitment by virus particles, remain to be determined. Such differences are likely to impact on the efficiency of virus-receptor engagement and the kinetics and outcome of virus fusion/entry events, not least in the very different environments encountered in cell-free infection versus direct cell-to-cell transmission. New SRI techniques, with the potential to image and count individual molecules, may provide new insights into the optimal properties of receptor molecules necessary for HIV binding, fusion and entry.

### 3.4 Receptor/co-receptor interactions and co-localisation

Stable receptor/co-receptor interactions may offer preferred binding sites for HIV by displaying both of the key molecules required for entry in close proximity. Alternatively, the assembly of a fusion complex composed of multiple receptor/co-receptor molecules may require timed engagement to ensure synchronous activation of several Env trimers. Biochemical studies, using immunoprecipitation (IP) from detergent lysates of cells, have suggested some level of constitutive CD4/CXCR4 interaction [247]. Similarly, CD4/CCR5 complexes have been co-IP'ed. from lysates of transfected NIH3T3 cells, as well as primary CD4^+ve^ T cells, macrophages and monocytes, with the first two CD4 Ig domains and the CCR5 second extracellular loop implicated in association [248]. However, co-IPs can be compromised by the formation of mixed micelles or over expression. Thus, complementary techniques that localise receptors in intact cells are essential.

Immuno-fluorescence imaging has suggested the presence of CD4/CXCR4 co-clusters on primary PBMCs [249]. By contrast, little overlap was reported for CD4 and CXCR4 on CD4^+^ T cells and macrophages analysed by confocal microscopy [250], as well as for CD4^+^ PBMCs and transfected HEK293 cells [251,252]. Similarly, a 3D fluorescence study that used live transfected HEK293T cells reported that CD4- and CCR5-FP fusions co-localised to the same cellular structures (including intracellular vesicles after ligand-induced internalisation) but were probably not constitutively associated at the PM [253]. CD4/CCR5 clusters were reported on PBMCs [249] and were also suggested by FRAP and FRET studies on transfected HEK293T cells [208,254]. However, other FRET studies, also using live transfected HEK293T cells, failed to see co-localised CD4- and CCR5-FP fusion proteins [255]. On T cells, and engineered HeLa and HEK293T cells, CD4- and CCR5-FP fusions have been seen to localise on microvilli and membrane ruffles, together with ezrin (and actin), however the microvilli-associated receptors did not appear to form stable complexes [253]. Most of the current morphological data derive from diffraction-limited fluorescence imaging, which is typically limited to spatial resolutions of 200–300 nm, >20 times the overall dimensions of individual receptor molecules. Again, complementary techniques with higher resolution, FRET and EM, can be helpful. Nevertheless, in all cases there is the potential for results to be influenced by experimental variables, including cell fixation, the use of cross-linking probes (e.g. Abs), variable activation states and culture conditions, over-expression in engineered cell lines, lack of effective quantitation, and different topological features (e.g. microvilli and plasma membrane ruffles that are not seen by fluorescence microscopy) that vary to a greater or lesser extent between different cell populations and lines. The future application of SRI, combined with automated analytical segmentation and quantitative analysis, will undoubtedly refine our current knowledge [176].

**Fig. 5:**
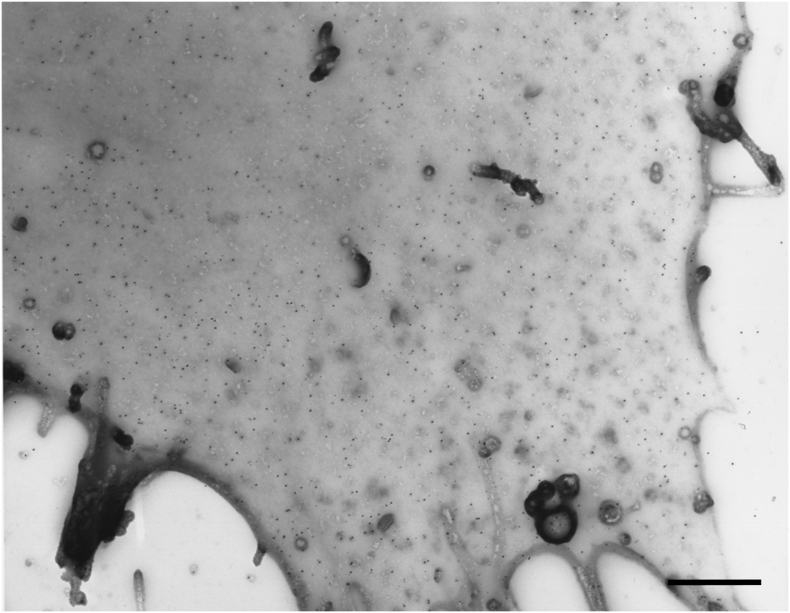
Electron micrograph of CD4 and CCR5 cell-surface distribution on Chinese hamster ovary (CHO) cells. CHO cells stably expressing human CCR5 and CD4 were fixed and labelled intact with an anti-CCR5 mAb (MC-5) followed by 15 nm diameter protein A gold, and with an anti-CD4 mAb followed by 10 nm diameter gold. Cell surface replicas were prepared as described in reference [199]. Scale bar: 500 nm.

Immuno-EM studies in primary T cells, macrophages and engineered HeLa cells reported preferential localisation of CD4, CCR5 and CXCR4 homo-clusters to PM protrusions (e.g. microvilli and blebs), with clusters of different molecules exhibiting low levels of co-localisation but often separated by distances <100 nm [256]. Similarly, an Lck-dependent localisation of CD4 to microvilli has been reported in CEM T cells [73]. Our own EM studies using whole mount EM, in which topological features can clearly be seen [199], indicated that CCR5 adopts a random distribution on stable transfected Chinese hamster ovary (CHO) cells but rapidly forms clathrin-associated clusters following treatment with agonist [199,176]. Significantly, on CHO cells expressing both human CD4 and CCR5, CD4 also adopted a dispersed distribution, in which there appeared to be occasional random close associations between CD4 and CCR5 (Marsh et al. unpublished, Fig. 5). Moreover, in mink lung fibroblasts transfected to express human CD4 and CCR5 or CXCR4, agonist-induced internalisation of CCR5 or CXCR4 did not result in CD4 co-internalisation [200].

## 4 Virus-cell interactions for virus entry

A general model for HIV entry into cells proposes that virus particles initially engage CD4 and subsequently CCR5 or CXCR4 molecules. HIV binding to CD4 and CCR5/CXCR4 leads to the redistribution and co-clustering of these molecules in the PM, likely through interactions with the actin cytoskeleton. HIV interaction with co-receptors leads to exposure of the gp41 fusion peptide and subsequent fusion of the viral membrane with the target cell membrane (Section 1.1). Adhesion proteins of various types, including integrins and heparin sulphate proteoglycans, can aid virus recruitment to cell surfaces prior to the engagement of specific receptors and endocytosis of bound virions may precede fusion in some cases. Trafficking of cell-associated viruses and/or receptor molecules, may be necessary to ensure effective receptor engagement, and the kinetics of receptor engagement may determine whether bound virions undergo fusion with the PM or endocytosis.

### 4.1 Receptor-virus binding

A number of studies have attempted to characterise the binding strength and lifetime for receptor/co-receptor-bound HIV Env gp120. Reports of measured equilibrium dissociation constants (*K*_d_) for gp120-CD4 and gp120-co-receptor binding from equilibrium binding assays have been reviewed [257,258]. *K*_d_ values for gp120-CD4 binding were in the range 1–300 nM [[259], [260], [261], [262], [263], [264],^39^]. Within this significant variability, higher *K*_d_ values (lower binding affinity) were measured for primary HIV isolates compared to lab strains adapted for growth in cell culture, as well as for intact virus particles compared to detergent-solubilised or recombinant soluble gp120, and for monovalent soluble CD4 (sCD4) compared to divalent sCD4-IgG chimeras [263]. For gp120-co-receptor interactions, the affinity for direct binding is low and the presence of CD4 is required to stabilise a gp120 conformation in which the co-receptor binding site is exposed [36,37,39,265]. *K*_d_ values measured for gp120-CCR5 binding in the presence of sCD4 were 4–6 nM (for various strains of Env) [39,257,266]. Env-CXCR4 binding is difficult to detect in equilibrium assays (due to the rapid off-rate) but a *K*_d_ of 500 nM was measured using a sensitive SPR assay [111]. However, as discussed above, ensemble average affinity values do not really describe the relevant receptor-virus binding interactions in virus entry, and single molecule force spectroscopy measurements, ideally in living cells, are preferable.

A few single molecule force spectroscopy studies have focused on gp120 binding to CD4 and CCR5, and on the role of the mechanical properties of CD4 in HIV entry. Binding of gp120 to individual CD4 and CCR5 molecules in living cells (e.g. CD4^+^ CCR5^−^ GHOST, CD4^−^ CCR5^+^ HOS and CD4^+^ CCR5^+^ GHOST Hi-5) has been probed by AFM [267,268]. Using an AFM cantilever tip functionalised with monomeric gp120, a rupture force for gp120-CD4 bonds of ~20 pN at 70 pN/s loading rate (pulling speed ~1 μm/s) was measured, 30 pN at 350 pN/s, 60 pN at 1000 pN/s and 110 pN at 3500 pN/s [267]. The predicted unstressed gp120-CD4 bond lifetime (at zero force) was ~0.24 s. For gp120-CCR5 bonds, rupture forces were ~20–30 pN at 90 pN/s loading rate, 40 pN at 500–600 pN/s and 80 pN at 3000 pN/s, an unstressed bond lifetime of 0.8–1.4 s was predicted, and the presence of CD4 (either soluble or membrane-associated) was required for gp120-CCR5 binding [267]. A further study by the same authors, using trimeric as opposed to monomeric gp120, measured similar gp120-CD4 bond-rupture forces [268]. Another AFM experiment reported gp120-sCD4 bond-rupture forces of 25 ± 20 pN on mica substrates, but loading rates were not given and the results varied significantly with tip and substrate functionalisation [269].

Together these data indicate that at low loading rates the forces required to break gp120-CD4 and gp120-CCR5 bonds were of similar magnitude (~20 pN), but gp120-CCR5 bonds had a significantly higher unstressed lifetime [267], suggesting that for gp120-CCR5 engagement to be possible within the short lifetime of a single gp120-CD4 bond, close proximity of CD4 and CCR5 would be necessary [267]. We estimate that such required proximity corresponds to a CD4-CCR5 distance <200 nm (calculated as the square root of the mean squared displacement (*msd*) for 2D Brownian diffusion, *msd* = 4*Dτ*, where we use a gp120-CD4 bond lifetime *τ*~0.24 s and a CCR5 diffusion coefficient *D*~0.04 μm^2^/s). Given that the mean distance between CD4 molecules on a T cells is ~100–200 nm and ~1000 nm for CCR5 molecules on average (both of which follow from the estimations presented in Section 3.1 using measured receptor numbers), it follows that a low fraction of CD4 molecules will find CCR5 molecules at distances <200 nm (whereas the majority of CCR5 molecules will have CD4 molecules within 200 nm distances), pointing to the likely necessity of multivalent gp120-CD4 interactions to extend the lifetime of the initial virus engagement, or to the possibility of active co-receptor transport mechanisms to guarantee co-receptor recruitment.

Two of these AFM studies suggested a possible conformational flexibility of gp120 [267,268]. This was inferred from two distinct slopes observed with low and high loading rates for the measured mean bond-rupture force as a function of force loading rate, indicative of intermediate conformations and energy barriers in the dissociation energy landscape [267]. Conformational changes in gp120 upon CD4 binding (to facilitate subsequent binding to co-receptors) were also inferred from the observed weakening and destabilisation of gp120-CD4 bonds with time (over 0.3 s), an effect enhanced by the presence of CCR5. The gp120-CCR5 bonds (in the presence of sCD4), on the other hand, were more stable and hardly changed over the same time scale [268].

The forces required to unfold the CD4 Ig domains are substantially higher than those required for rupturing gp120-CD4 bonds. The role of the mechanical properties of CD4 in its binding interactions with HIV Env has been investigated by unfolding Ig-like domains 1 and 2 (D1 and D2) [270]. AFM was used to pull CD4 D1D2 complexes immobilised on a gold surface. From measurements at constant pulling speed, the unfolding forces for D1 and D2 were approx. 60 pN and 80 pN, respectively, at low pulling speeds (~10 nm/s), with higher unfolding forces measured at higher pulling speeds. D2 often unfolded before D1, possibly owing to the presence of an allosteric disulphide bond in D2 [271]. The fingerprint extension lengths upon unfolding for D1 and D2 were of the order of 10 nm. Measurements at multiple constant forces predicted a non-zero unfolding rate at zero force (by extrapolation), implying that mechanical extension of these CD4 domains can take place even at very low forces, such as those that are potentially involved in cell-free virus entry. The authors also correlated increased levels of CD4-domain extension with increased HIV infectivity and measured how Ibalizumab, an HIV-neutralizing anti-CD4 mAb, reduced infectivity by increasing the mechanical stiffness of CD4 molecules. Similar unfolding forces may be expected for the D3 and D4 domains of CD4, given their structural similarity to D1 and D2, as shown by computational calculations in the same study [270], although D3 does not have a disulphide bond which might make it unfold at lower pulling forces.

Another AFM study of CD4 unfolding on mica substrates at a constant pulling rate (~1 μm/s) resulted in a mean rupture force ~150 pN, over a total displacement (pulling length) of ~120 nm before the AFM tip detached, suggesting the unfolding of several CD4 domains [272]. Single gp120 molecules on mica and gp120 aggregates adsorbed onto lipid bilayers displayed unfolding rupture forces and elongations similar to those for CD4 on mica, with gp120 displaying an ability to insert or sink into the lipid bilayer over time (in the absence of the transmembrane gp41 segment) [272].

It is important to note that molecular unbinding/unfolding depend not only on the force applied but also on the dynamic pulling rate. Additionally, in living cells, dynamic events such as receptor clustering or conformational changes after signalling, can result in altered binding kinetics. Furthermore, ligand binding/unbinding rates for cell-surface receptors can be very different in isolated cells in solution, compared to cells exposed to forces and interactions such as those arising, for example, from fluid flow or from close cell packing in lymphoid tissue. External forces exerted on the virus during cell-free virus interaction in solution are expected to be low [267]. However, forces present during cell-to-cell virus transfer might be substantially higher.

Currently, we know little about the numbers of Env-receptor/co-receptor bonds involved in virus-cell binding or the possible cooperative effect of receptor/co-receptor clustering on bond lifetime and strength upon viral engagement. Similarly, little is known about receptors/co-receptor attachments to the actin cytoskeleton and possible changes in these interactions upon HIV engagement. HIV binding leads to the redistribution and co-clustering of CD4 and CCR5/CXCR4 molecules in the PM (Section 4.4), which may be aided through links to the actin cytoskeleton. Force spectroscopy measurements to probe cytoskeleton links to cell-surface adhesion molecules have been reported [121]. Similar force spectroscopy measurements of receptor-cytoskeleton links before and after virus engagement would provide useful information. As an indication of the strength of F-actin attachment to actin-binding proteins, rupture forces of 40–80 pN have been measured at ~4–50 pN/s loading rates for filamin and alpha-actinin in vitro with optical tweezers [273], somewhat higher than those for gp120-CD4 and sCD4/gp120-CCR5 bonds at comparable loading rates. Another parameter for which little is known is the magnitude of the forces required for virus internalisation via endocytosis. For instance, average peak forces of ~4 pN have been measured during the initial pulling events involved in clathrin-mediated endocytosis of epidermal growth factor (EGF)/EGF receptor complexes [137]. The forces required to create substantial membrane deformation (endocytosis or pulling elongated tubes) in mammalian cells are in the order of tens of pN or higher [274,275]. Endocytosis of full virus particles is likely to involve equivalent forces.

In summary, there is substantial room for further investigations into the molecular interactions, forces and mechanical properties relevant to HIV entry, using both well-established and novel force-sensing techniques. Combined force sensing and fluorescence detection may be especially useful in these studies [[276], [277], [278]]. A broader application of quantitative force spectroscopy and unbinding measurements in living cells may be extremely useful for comparing different virus strains and to assess the effects of various antiviral drugs [279].

### 4.2 Virus movements on the plasma membrane prior to entry

Viruses can display various patterns of motion on the cell surface prior to entry into a cell [[165], [166], [167],^280^,^281^], with different mobility modes reflecting distinct entry and transmission routes (e.g. initial penetration through mucosal/epithelial barriers, cell-to-cell transfer or cell-free infection). These mobility modes include: (i) fast, random diffusion of viruses on the PM (over μm length scales and second time scales; similar to the diffusion rates measured for CD4 and CCR5 [Section 3.2]) that can facilitate the recruitment of cell-surface receptors and/or signalling proteins and/or the encounter of entry sites on the membrane; (ii) directed motion on the PM mediated by coupling to myosin-driven filamentous actin, e.g. surfing to enable viral transmission between cells; and (iii) local confinement of viruses to sub-μm sized areas to facilitate viral uptake via endocytosis. Sliding and tumbling motions during lateral diffusion and back and forth rocking have also been observed for viruses other than HIV [282]. These events have been detected by fluorescence microscopy of tagged viruses and SPT analysis.

For HIV, surfing of virions (YFP-Gag labelled) bound to filopodial membrane protrusions has been observed in HEK293 cells expressing CD4 and CXCR4 [165]. Virus surfing along filopodia or microvilli can be particularly relevant to virus infection via the penetration of microvilli-rich mucosal surfaces and epithelial barriers, with mucosal transmission thought to account for the majority of in vivo primary HIV infections [165]. Filopodial bridges and interdigitated membrane surfaces may also operate within HIV virological synapses, as revealed by recent 3D imaging [283], and ‘surfing’ of receptor-associated HIV virions along filopodial bridges has been observed and proposed to mediate efficient cell-to-cell transmission [166].

As some of these patterns of virus motion may be driven by host cell cytoskeletal dynamics, it is important to have a better understanding of how virus-cytoskeleton links are established and regulated, and how HIV receptor/co-receptor diffusion relates to observed patterns of virus motion.

### 4.3 Env in the viral membrane

As with receptors in the PM, the mobility of Env and lipid molecules in the viral membrane can also influence the virus-cell interactions leading to virus entry. HIV virions generally carry 10–14 Env trimers. Though diffusely distributed on immature viruses, these molecules are frequently clustered on the surface of mature virions [7,14,15,168]. Clustering of Env trimers on the virus surface can contribute to the clustering of HIV receptors/co-receptors observed in the PM on virus binding. SRI has indicated that Env clustering accompanies virus maturation, suggesting 1) that Env mobility is restricted by intact p55 Gag molecules and 2) that Env molecules randomly distributed on immature virions are not able to induce fusion [168].

Published work has shown that HIV-1 infectivity correlates with Env density, with a reduction in the number of trimers leading to a decreased efficiency of viral infectivity [284,285]. Initial findings suggested that a minimum number of functional Env trimers is required to mediate fusion [284,286], but the number of trimers required (referred to as HIV entry stoichiometry) has been controversial, with estimated values ranging from 1 to ~19 [284,[286], [287], [288], [289], [290], [291], [292], [293], [294], [295]]. It is important to note that measurements are not direct and the results uncertain, with data interpretation being complex and relying significantly on mathematical model assumptions [294,292,293]. Some of the initial lower estimates (~1 Env trimer) [[287], [288], [289]] resulted from models that did not consider multiple trimers per virion and hence favoured low estimates of HIV entry stoichiometry [293,294]. Later analysis of the same data (data from [287]) with alternative underlying mathematical models suggested higher numbers of 4–8 Env trimers [290,291]. More recently, it has been shown that the number of Env trimers required for entry varies for different HIV strains, with most strains requiring 2–3 trimers (the full range was 1–7 trimers), and that strains with higher entry stoichiometry had lower virus infectivity and slower entry kinetics [295].

The lipid requirements for HIV fusion are not well established: it has been proposed that cholesterol is required and that the virus can sense and exploit discontinuities between liquid ordered and disordered domains for fusion [223]. Whether similar discontinuities occur or are required for HIV fusion in endosomes is unclear.

### 4.4 Receptor and co-receptor redistribution and clustering upon viral engagement

It is unclear whether specific numbers of Env-CD4 and/or Env-CCR5/CXCR4 interactions are required for HIV entry. Likewise, the stoichiometry and exact mechanism of HIV receptor and co-receptor clustering remain obscure, and we lack data for the dynamics of HIV receptor clustering. HIV binding can trigger the redistribution and clustering of PM CD4 and co-receptors to sites of HIV gp120 binding, as revealed by several confocal immunofluorescence microscopy studies [86,88,[250], [251], [252],^255^,^296^]. For instance, gp120-CD4 engagement on HEK293-CD4 cells and PBMCs can trigger the lateral diffusion and co-clustering of gp120-bound CD4 with CXCR4 and with raft-like micro-domains [251]. Similar clustering has been observed in T cells using intact HIV via SRI [297]. Clustering events can greatly enhance the probability of the molecular interactions required for virus entry occurring, i.e. molecular orientation and cooperative binding may be optimised in receptor clusters and the mobility of the clusters will be reduced compared to that of individual receptors [100,298].

It is unclear whether CD4 binding alone is sufficient to trigger the redistribution and clustering of CCR5 and/or CXCR4 and F-actin. Using fluorescence microscopy, it has been observed that CD4 binding by anti-CD4-coated micro-beads can trigger the redistribution of CCR5 and CXCR4 (and of lipid-raft components and adhesion molecules) to sites of bead contact with the surface of human T cells [296]. These rearrangements require F-actin polymerisation, Lck signalling and cholesterol. However, other work has suggested that CD4 engagement alone is not sufficient to trigger co-receptor redistribution and that co-receptor engagement and signalling are required to generate the cytoskeletal rearrangements necessary for productive virus entry [88].

### 4.5 Receptor-cytoskeleton links and molecular signalling pathways for HIV entry

Recent studies have shown that clustering is mediated by cytoskeleton rearrangements and regulated by a number of actin-binding and adaptor proteins that, directly or indirectly, link receptors/co-receptors to the actin cytoskeleton. Various molecular complexes are known to participate in the early signalling pathways that lead to HIV entry, as summarised in Table 1. Together the data suggest the following model: initial engagement of gp120 would trigger the activation, with the involvement of Lck [78], of the actin-binding adaptor proteins, filamin-A, moesin and ezrin, to establish links between CD4, co-receptors and F-actin, regulating receptor/co-receptor clustering and redistribution in the PM [88,86,87]. These events would require cortical actin cytoskeleton dynamics regulated by actin-related proteins gelsolin, syntenin-1, drebrin and cofilin [[96], [97], [98],^95^,^20^]. Other signalling events contributing to the cytoskeletal rearrangements necessary for viral entry would be the activation of the RhoA/ROCK/LIMK/cofilin signalling pathway, which would again require filamin-A. This would lead to the initial inactivation of the actin-severing protein cofilin, and to the activation of the interleukin-2-inducible T-cell kinase (ITK) and of the GTPase Rac-1 via the chemokine co-receptors [[92], [93], [94]]. Signalling through the co-receptors (CXCR4) would subsequently result in transient cofilin activation and F-actin severing to remove the cortical actin barrier and enable viral entry [20]. There is also evidence that gp120-co-receptor interactions trigger signalling cascades that result in the exposure of the membrane lipid phosphatidylserine (PS) on the cell surface, with PS-Env interactions facilitating virus fusion and entry [21].

### 4.6 HIV virological synapses

As previously mentioned, cell-to-cell transmission via virological synapses may be particularly relevant to infection events in vivo. In this respect, the clustering and molecular binding interactions in VS are likely to be substantially different to those that virions encounter during cell-free infection. For example, TCR-MHC binding in T-cell immunological synapses (IS) exhibits higher binding affinities resulting from ~100-fold higher association rates and ~10-fold faster dissociation rates compared to affinities measured in 3D solution assays [299,300].

Currently, our knowledge of VS is limited. Little is known about the role of filamin-A, the ERM proteins, cofilin and Lck in VS [301]. For instance, activated ezrin has been proposed to prevent cell-cell fusion in early-stage VS, a feature that is likely to be important for HIV transmission, but how this occurs is not understood [302]. Env-induced activation of Lck in artificial VS has been linked to the formation of an actin-depleted zone at the centre of the VS and may be related to actin-reorganisation events seen in IS formation [79]. Indeed, there are significant parallels between HIV VS and T-cell IS [301,303]. In both cases, immune cells and target cells form close contacts (synapses), to which key proteins are recruited and accumulated to assist in virus cell-to-cell transfer, or lymphocyte activation, respectively. Similar to how cellular adhesion is secured in IS [304], in HIV VS the cell contact region is stabilised by intercellular adhesion molecules (e.g. ICAM-1 and -3) on the infected cell and the integrin receptor LFA-1 on the target cell, with actin-binding proteins α-actinin and talin recruited to the synapse possibly to link these adhesion proteins to the actin cytoskeleton [301]. The formation of a central cluster of receptors at the centre of the synapse, with the recruitment of CD4, CXCR4/CCR5 and TCRs, and the possible involvement of Lck and TCR signalling and cofilin, are other examples of features common to both VS and IS [88,79,301,305,306]. These shared features suggest that common processes mediate protein recruitment in both cases. Further details of HIV cell-to-cell transmission in VS can be found in several recent reviews [54,55,[307], [308], [309], [310]]. In general, it remains unclear whether the role of proteins relevant for cell-free infection is the same for VS-mediated transmission. Approaches such as BFP micropipette assays in combination with light-sheet fluorescence microscopy are best placed to characterise single molecular events in synaptic interactions.

## 5 Models and simulations

The measurements discussed above provide some insight to the biological and physical processes that influence the efficiency of virus entry. These include the density, distribution, diffusive mobility and clustering of receptors and co-receptors in the PM, virus-receptor binding and unbinding rates, and stoichiometries, and how these are influenced by external forces applied to the virus-receptor bonds and by thermal Brownian motion. All of these aspects can be integrated into computational simulations with the aim of generating predictions as to the features most crucial for productive virus entry.

One such model simulated HIV entry by considering static viruses in the vicinity of freely diffusing receptors [311]. A single receptor species was considered (without distinction between receptors and co-receptors) with a given diffusion coefficient and receptor concentration. The virus-receptor binding and unbinding rates were set, as well as a virus degradation/detachment rate. The model assumed virus entry occurred when a virus was bound by a threshold number (N) of receptors. The entry probability was found to increase with receptor mobility. For highly mobile receptors, the entry probability increased with N, whereas this trend was inverted for less mobile receptors [311].

A Brownian dynamics model accounting for receptor concentration and diffusion on the cell surface as well as for Env density on the virus surface showed initial engagement of CD4 by Env trimers taking place within fractions of a second, to tether the virus to the cell surface. Subsequent accumulation of CD4 molecules in the virus-cell contact area followed within a few seconds, driven by random diffusion (no receptor-cytoskeleton interactions were considered) and resulting in the engagement of other Env trimers to strengthen binding. Although the Env trimers were randomly distributed on the viral surface in this model, and not allowed to diffuse, the virus-cell binding rates were comparable for viruses with differing numbers of Env trimers, from as few as 8 to as many as 72 [312].

Another stochastic model predicted the formation of organised cell-virus adhesion interfaces with ring-like nano-structures of gp120-bound CD4 and CCR5 molecules, similar to IS but at a smaller length scale ~50 nm [313]. HIV was modelled as a mobile, rigid sphere with Env trimers capable of binding up to three CD4 and three CCR5 molecules each. Receptors in the PM were given experimentally determined 2D diffusion rates and surface densities (with a CD4:CCR5 ratio of 10:1). The authors used data for gp120-CD4 and gp120-CCR5 binding/unbinding rates from force spectroscopy experiments [268]. When the model allowed Env trimers to diffuse on the virus surface (with 15 gp120 trimers per virion), the junction evolved over a time of ~200 ms from a single bound trimer to a steady-state number of multiple trimers bound to CD4 and CCR5 molecules organised into a ring-like structure (up to ~25 CD4-gp120 bonds and ~3 CCR5-gp120 bonds). PM deformation and sinking of the virus into the PM (over ~20 nm) took place over a time scale of ~3 s. Increased plasma membrane rigidity led to reduced virus-sinking depths as well as to increased CD4-gp120 bond numbers, pointing to the importance of the PM mechanical properties in virus entry [313]. Modelling viruses as having an even distribution of immobile Env trimers instead, a 3-fold lower steady-state number of CD4-Env bonds was formed over a 10-fold longer time [similarly to the previous model [312]], while there was no significant changes in the number of CCR5-gp120 bonds or in the time scale of PM deformation and virus sinking. No ring-like structure or PM deformation were observed for low numbers (<9) of fixed Env trimers per virion [313].

New precise measurements of the dynamics of HIV entry, including HIV-receptor redistribution and clustering at virus-binding sites, should allow testing of the above-mentioned models with the aim of deciphering the exact biophysical mechanisms driving receptor recruitment and virus penetration. However, it remains a challenge to include poorly understood dynamic parameters into models, e.g. dynamic changes in receptor concentration and mobility, the existence of different populations of receptors with different diffusive behaviours or aggregation states, and the influence of all relevant molecular interactions (e.g. receptors with co-receptors, or receptors/co-receptors with F-actin). However, robust experimental measurements will provide information to improve the value of further modelling efforts.

## 6 Conclusions

Research from many labs has provided considerable insight into the mechanism of HIV entry. Nevertheless, it is clear that we lack a detailed molecular view of the properties of the HIV receptors, and how these molecules are engaged during virus entry. The extent of receptor oligomerisation and receptor/co-receptor co-localisation in particular are contradictory, as are measurements of diffusion. Whether or not these differences are due to the use of different cell lines, or experimental conditions, is unclear, but the need for a systematic study is obvious.

For this, a set of clear conditions needs to be established. It is apparent that measurements of receptor properties can be influenced by glass surfaces, the nature of the ligands, the cellular background and topology, and environmental conditions such as temperature. We would advocate that high temporal resolution SPT (as opposed to FRAP) at 37 °C is one of the best techniques to measure receptor mobility as the analysis of individual receptor trajectories enables the efficient classification of subpopulations with different diffusive behaviours. Alternatively, FCS in combination with STED SRI enables simultaneous high spatial and high temporal resolution measurements of protein and lipid mobility in the PM of living cells [184,314]. With attention to this set of parameters, it should be possible to generate data sets that are reliable and comparable. Fluorescence SRI in particular will prove extremely useful for refining our current knowledge. With these approaches, not only should existing data be revisited, but currently open questions should be addressed, e.g. how does virus engagement influence the properties of CD4 and CCR5/CXCR4, do CCR5 and CXCR4 exhibit distinct properties (we have virtually no data on CXCR4), how does the actin cytoskeleton and its interaction with the HIV receptors/co-receptors influence HIV entry, how does clustering affect receptor mobility and virus-receptor binding, what are the numbers of Env and receptor/co-receptor molecules and bonds required for productive entry?

Nevertheless, the available data do indicate some clear trends. CD4 and CCR5 seem to have similar diffusive mobilities in the PM of cells and gp120-CD4 and gp120-CCR5 single molecular bonds are short lived (~0.2 s and ~1 s lifetimes, respectively) compared to the time scale of virus entry events, which is of the order of minutes (from engagement to fusion). Hence, receptor-virus interactions will necessarily be multivalent to guarantee a sufficiently long engagement duration, as multivalent bond lifetimes increase exponentially with the number of binding partners [315]. In light of this, receptor and co-receptor densities in the PM and their ratios will determine the probability of the necessary molecular encounters required for virus entry occurring. Human primary CD4^+^ T cells have ~4 times more CD4 molecules than co-receptor molecules on their surface (on average) whereas this ratio can vary substantially in cell lines. These high CD4 numbers (and possibly high CD4 densities) are likely crucial for robust initial engagement and tethering of HIV to the cell surface, with CD4 clustering and the trimeric nature of Env-gp120 likely guaranteeing that, despite the short lifetime of gp120-CD4 individual bonds, gp120-CD4 interactions are sustained for long enough to allow the conformational changes that lead to co-receptor engagement to occur. As gp120-CCR5 bonds are longer lived, viruses can afford the lower co-receptor numbers (and likely lower co-receptor densities) in the PM, but only to a certain extent. Co-receptor number/density may be crucial for triggering signalling activities required for virus entry, with a threshold density of co-receptors in the PM likely to be required for efficient infection and increases in CCR5 levels leading to increased entry and subsequent virus production.

Importantly, the literature we have reviewed focuses largely on available data acquired from monolayer (two-dimensional) cell cultures. Further data with higher physiological relevance may one day be acquired via in vivo animal imaging or by application of recent advances in alternative, three-dimensional cell-culture systems [316,317] (e.g. lymphoid tissue and organoids generated from induced pluripotent stem cells).

The viable prospect of applying a number of new techniques to the various aspects of virus entry discussed in this review, and to complement these experimental approaches with integrative theoretical models and computational simulations, has the potential to provide insight to the biophysical principles that underlie HIV entry specifically, but should also be applicable to a variety of other viruses.

## Transparency document

Transparency document. Image 1

## Declaration of competing interest

The authors declare that they have no known competing financial interests or personal relationships that could have appeared to influence the work reported in this paper.
